# A quantitative approach to study indirect effects among disease proteins in the human protein interaction network

**DOI:** 10.1186/1752-0509-4-103

**Published:** 2010-07-29

**Authors:** Thanh-Phuong Nguyen, Ferenc Jordán

**Affiliations:** 1 The Microsoft Research - University of Trento, Centre for Computational and Systems Biology, Piazza Manci 17, 38100, Trento, Italy

## Abstract

**Background:**

Systems biology makes it possible to study larger and more intricate systems than before, so it is now possible to look at the molecular basis of several diseases in parallel. Analyzing the interaction network of proteins in the cell can be the key to understand how complex processes lead to diseases. Novel tools in network analysis provide the possibility to quantify the key interacting proteins in large networks as well as proteins that connect them. Here we suggest a new method to study the relationships between topology and functionality of the protein-protein interaction network, by identifying key mediator proteins possibly maintaining indirect relationships among proteins causing various diseases.

**Results:**

Based on the i2d and OMIM databases, we have constructed (i) a network of proteins causing five selected diseases (DP, disease proteins) plus their interacting partners (IP, non-disease proteins), the DPIP network and (ii) a protein network showing only these IPs and their interactions, the IP network. The five investigated diseases were (1) various cancers, (2) heart diseases, (3) obesity, (4) diabetes and (5) autism. We have quantified the number and strength of IP-mediated indirect effects between the five groups of disease proteins and hypothetically identified the most important mediator proteins linking heart disease to obesity or diabetes in the IP network. The results present the relationship between mediator role and centrality, as well as between mediator role and functional properties of these proteins.

**Conclusions:**

We show that a protein which plays an important indirect mediator role between two diseases is not necessarily a hub in the PPI network. This may suggest that, even if hub proteins and disease proteins are trivially of great interest, mediators may also deserve more attention, especially if disease-disease associations are to be understood. Identifying the hubs may not be sufficient to understand particular pathways. We have found that the mediators between heart diseases and obesity, as well as heart diseases and diabetes are of relatively high functional importance in the cell. The mediator proteins suggested here should be experimentally tested as products of hypothetical disease-related proteins.

## Background

The information in the human genome is sometimes so crucial that simple changes can lead to severe inheritable or chronic diseases. Genes related to causing diseases are called disease genes [1] and their protein products are disease proteins. Traditional biological and medical methods to study those genes may require expensive and laborious experiments. Thus there is a great need to develop alternative (e.g., computational) methods to understand them.

There is massive research on discovering disease genes with various methods and data sources. Early works on disease genes were based on either annotations (e.g. [2]) or sequence analysis (e.g. [3]). Generally, these methods considered disease genes separately, as if they were independent. However, we increasingly recognize that biological processes are typically driven by the interplay of complex interaction networks of various molecules (mostly proteins). Not surprisingly, many human diseases can be traced to aberrant protein-protein interactions, either through the loss of an essential interaction or through the formation of a protein complex at an inappropriate time or location [4,5]. Therefore, it is encouraging and promising to study disease genes in the context of the complete protein interaction network.

Research on protein-protein interaction networks (PPI network) and diseases has been much appealing in recent years (see [6] for the k-nearest neighbor algorithm, [7] for the phenomic ranking of protein complexes and [8] for combining gene expression and PPI, and [9] for the semi-supervised learning approach with multiple data sources). Also, there are several work that concentrated on studying disease genes for only one specific disease (e. g. Alzheimer disease) using heuristic score functions [10,11]. Key reviews on computational methods for disease genes are provided by Kann [12] and Ideker and Sharan [13].

In previous work, the direct associations of disease proteins were brought into focus to predict disease proteins. However, in complex diseases such as the ones studied here, the rules that underlie the pathogenesis are not only mutations in a single protein, but result from complex interplay among them in the whole network and genome-environment interactions. Consequently, the association among proteins causing disease is much more intricate. They may not directly affect each other to cause disease, but indirectly, through a mediator or a group of mediators.

As bioinformatics and systems biology offers large databases and novel computational tools for better understanding complex systems, we approach the disease-gene problem in a different way. Even if now it is possible to quantify complexity and take a quantitative, holistic approach, it is not yet easy to really understand the indirect determination of biological processes. Here graph theory and network analysis can be helpful. Network analysis provides information about the local (node-level) and global (whole graph-level) properties of the system. For example, the degree distribution of a complex network is a property that is global but based on local, limited information on the positions of nodes (reflecting only to neighbors, the rest of the network is simply not considered while characterizing a given node). However, there are a number of more sophisticated network analytical tools to explicitly measure indirect relationships (for example, see [14,15]).

In this paper, we propose a new network analytical method to quantify indirect effects among proteins in their interaction network by using network indices [16,17] and illustrate its use for studying PPI networks. In particular, our aim is to quantify the indirect relationships among proteins involved in five diseases, and to characterize the proteins mediating these indirect effects. The five diseases are cancer (C), diabetes (D), obesity (O), heart diseases (H) and autism (A). The first four ones belong to the major threats and mostly studied disorders. Autism, on the contrary, is a relatively minor disease. The autism genes were studied in this work, since it seemed to be an interesting question how a systems-based approach can or cannot reveal direct and/or indirect effects between seemingly independent diseases [18]. In addition, based on the number of scientific publications, there is a fastest-growing interest in autism and discovering possible cross-links can only be a matter of time and effort. As it was earlier suggested that functionally more important proteins are hubs and disease proteins are peripheral ones in protein networks [19], our interest is focused on whether the hubs are mostly responsible for connecting these disease proteins to each other. We concentrated on identifying the key "mediator proteins" defined as non-disease proteins that are interactive partners (IPs) of disease proteins in the PPI network. We note here that a mediator protein may well be considered a hypothetical disease protein which is promisingly validated in wet-lab.

## Results

**Figure 1:**
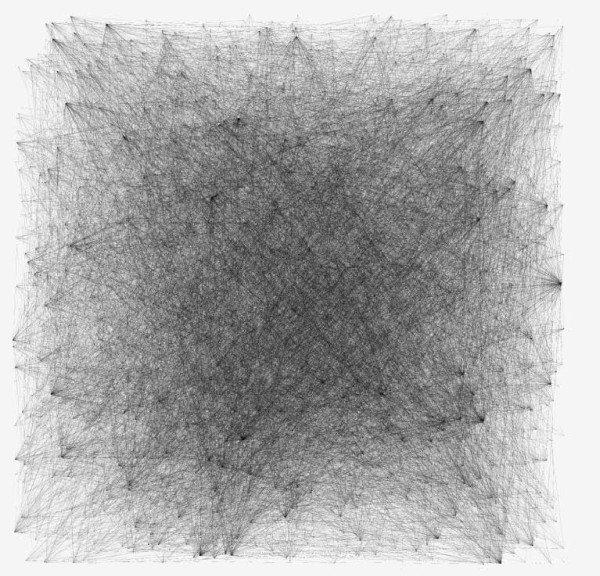
**The DPIP network**. The figure shows the DPIP network of our study. It contains disease proteins and their interacting partners (IPs). All hubs are cancer-related proteins. Figure drawn by [38].

**Figure 2:**
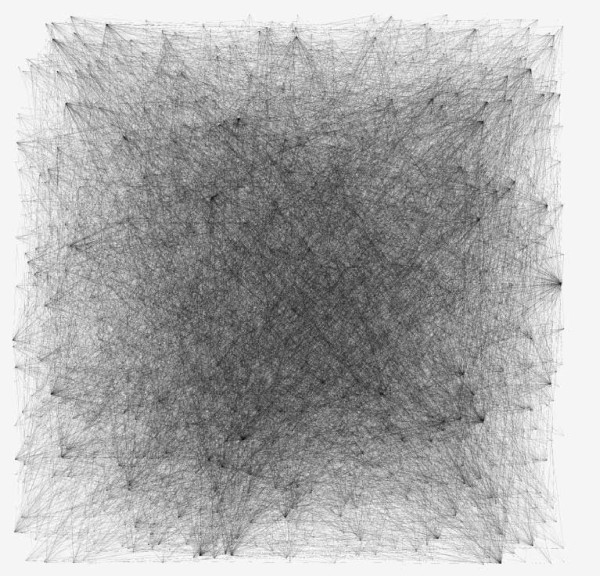
**The IP network**. The figure shows the IP network analyzed. It contains only IPs and the links among them. Figure drawn by [38].

Figure 1 shows the DPIP, while Figure 2 shows the IP network analyzed. In the DPIP network, two heart disease proteins (P16671 and P17302) are directly linked to a cancer protein (P12931) but no H proteins are linked to A, O or D proteins. Among the IP proteins connecting the otherwise unlinked sets of H and O proteins, P12931(proto-oncogene tyrosine-protein kinase Src) was the only non-IP protein mediating any effect. The most influenced H protein was P18825 and the most influenced O protein was P07550 (beta-2 adrenergic receptor). The strongest indirect effect was mediated by Q14232 (translation initiation factor eIF-2B subunit alpha) (see Table 1 for mediator identity and Table 2 for relative strength of effects).

**Table 1:** Proteins mediating between H and O proteins.

O\H	P08254	P08588	P16671	P17302	P18825	P78504	Q14524	Q9UGJ0	Q9Y4J8
O00253									

O75056			**P06241**	**P41240**					

P01189									

P07550		**P49407 P62993 Q5JY77**	**P12931**	**P12931 P17252 P41240**	**Q14232**				

P13945			**P12931**	**P12931**					

P25874									

P29120									

P32245									

P37231				**P28482**					

P41159								**P54646**	

P41968									

P48357									

P52895									

P55851					**P63104**				

P55916					**P63104**				

P81133									

Q15466									

Q16620		**O14908**	**P06241**						

Q86YN6									

Q9UBU3									

Identity of mediator proteins among H proteins causing heart diseases (in columns) and O proteins causing obesity (in rows). Most of the mediators are IP proteins, there is a single disease protein connecting the other two sets of disease proteins (P12931, belonging to C).

**Table 2:** Strengths of indirect effects between H and O proteins.

O\H	P08254	P08588	P16671	P17302	P18825	P78504	Q14524	Q9UGJ0	Q9Y4J8
O00253									

O75056			**0.0154**	**0.0155**					

P01189									

P07550		**0.0867**	**0.0005**	**0.0222**	**0.123**				

P13945			**0.0013**	**0.0013**					

P25874									

P29120									

P32245									

P37231				**0.0071**					

P41159								**0.0508**	

P41968									

P48357									

P52895									

P55851					**0.0583**				

P55916					**0.0583**				

P81133									

Q15466									

Q16620		**0.0575**	**0.012**						

Q86YN6									

Q9UBU3									

The relative strength of two-step-long indirect interactions (A_H, O_^2^) mediated by mediators among heart disease and obesity. Column sums would give which H protein is mostly influenced by O proteins (P18825), while row sums would give which O protein is mostly influenced by H proteins (P07550). The largest value (0.123) indicates the strongest indirect effect between the two diseases (corresponding to Q14232, see Table 1).

The same analysis for the mediators between the H and D sets of disease proteins (see Additional files 1 and 2) shows that there are eight shared mediators appearing in both links (H-O and H-D) but typically these shared mediators are less important in both systems (the exception is P63104 (14-3-3 protein zeta/delta) being among the most important mediators in both H-O and H-D pathways, marked by red in Additional file 3). Here, P12931 was again the only non-IP mediator, the most influenced H protein was P18825 (alpha-2C adrenergic receptor) again and the most influenced D protein was P51681 (C-C chemokine receptor type 5). The strongest indirect effect was mediated by P09471 (guanine nucleotide-binding protein G(o) subunit alpha).

We have determined the contribution of the other four sets of disease proteins C, D, O, and A to influencing the set of H proteins. By network analysis, it is possible to determine where the largest indirect effects can be expected from. For example, in case of the P18825 H protein, although there are more than twice as much O proteins than D proteins in this database, the indirect effects reaching H proteins from D proteins are stronger (39% of influence) than the ones originating at O proteins (26% of influence). Based on the sheer number of proteins, we would expect the contrary. Also, even if proteins linked to autism (A) are apparently independent of the rest of diseases, the strength of their topological relationship is comparable to other diseases (5% of influence). Cancer proteins, even if there are a lot of them, contribute only to 30% of influence on P18825.

Additional file 4 shows the hubs in the PPI and IP networks (measured by nD and nB). Additional file 3 shows the most important mediators between H and O as well as H and D disease proteins in the DPIP network (measured by M^2^). Some proteins are always of key importance, like P63104, that is a hub and a key mediator. Others, like Q9Y4K3 (TNF receptor-associated factor 6) are hubs in the PPI network (high nD) and highly central nodes in the IP network (high nB), but even so, they are not mediating indirect effects between the studied diseases.

**Figure 3:**
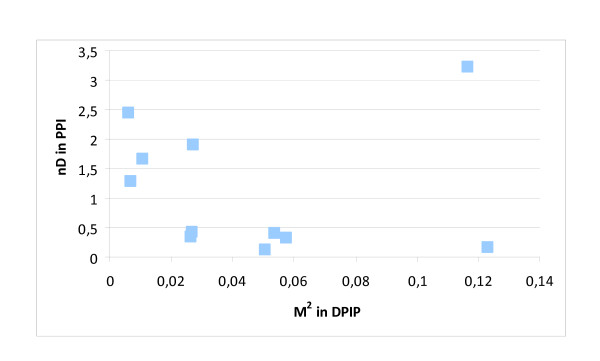
**Degree and H/O mediator role**. The importance of mediator proteins between H and O disease proteins (measured by M^2 ^in the DPIP network) is shown versus their nD in the PPI network. Important mediators among disease proteins are not necessarily hub proteins in the cell.

**Figure 4:**
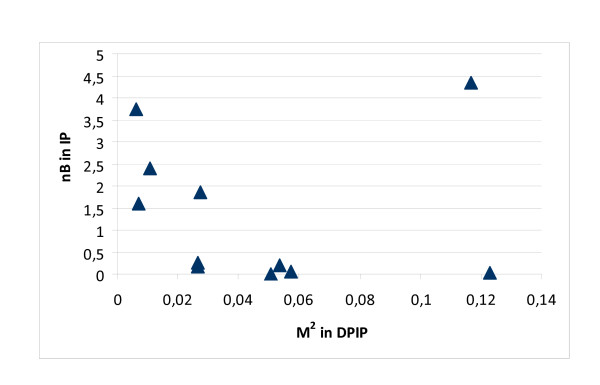
**Betweenness and H/O mediator role**. The importance of mediator proteins between H and O disease proteins (measured by M^2 ^in the DPIP network) is shown versus their nB in the IP network. Important mediators are not necessarily high-betweenness nodes in IP.

Figure 3 shows that important mediators (high M^2 ^in DPIP) are not necessarily hubs (high nD in PPI), but they can be (see the outlier point representing P63104). Similarly, Figure 4 shows that high-betweenness nodes in the IP network are not necessarily the key mediators among groups of disease proteins. These results are qualitatively similar in the symmetrical cases (nD in IP and nB in IP). Based on Figures 3 and 4, we can see that important mediator proteins may have only a few neighbors.

**Figure 5:**
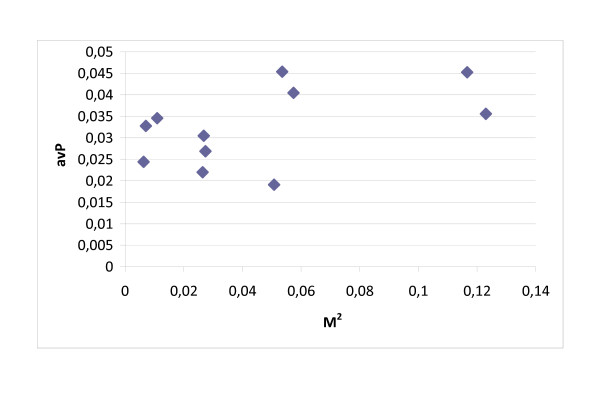
**Function and H/O mediator role**. The figure shows the average P-value (avP) of GO terms against the importance of the 11 most important mediator proteins (measured by M^2^) in H-O pathways. The P-value quantifies the significance of GO term enrichment with a modified Fisher's exact test.

**Figure 6:**
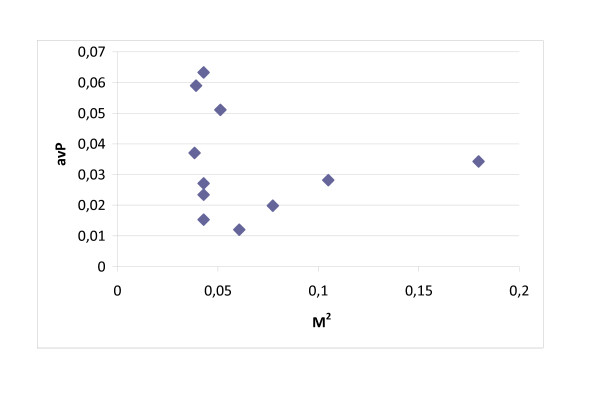
**Function and H/D mediator role**. The figure also shows the average P-value (avP) of GO terms against the importance of the 11 most important mediator proteins (measured by M^2^) in H-D pathways. The P-value quantifies the significance of GO term enrichment with a modified Fisher's exact test.

We suggest that it can be of interest to study the relationship between network position and functionality of proteins in the protein interaction network, even if our work indeed did not intend to predict causing-disease genes. Table 3 shows the statistics of enriched terms for the information of interest. The relationship between the M^2 ^index and the average P-value calculated for GO terms of the mediators is demonstrated for both the H-O (Figure 5) and the H-D (Figure 6) pathways. All of the P-value of GO terms for the top mediators are below 0.05 (except for the H-D mediators P80098 (C-C motif chemokine 7) and P22681 (E3 ubiquitin-protein ligase CBL)): it means that these mediators associate to rich GO terms. The Pearson correlation (ρ_H-O _= 0.51) and the Spearman rank order correlation (ρ = 0.55) show that there is a relatively strong positive relationship between M^2 ^and P-value of GO terms of the H-O mediators. For the H-D mediators, the relationship is negative and not very strong (with Pearson correlation ρ_H-D _= -0.14 and the Spearman rank order correlation ρ = -0.32). The relationship between important mediator function (high M^2^) and functional importance (low average P-value) is stronger in the case of diabetes (Figure 6).

**Table 3:** Functional statistics of mediator proteins.

	GO term	Pathway	Tissue	Domain
H-O mediator	67	49	73	90

H-D mediator	70	2	24	16

Statistics of data/terms regarding the GO terms, pathway, tissue, and domain information on the most important mediator proteins in the heart-obesity (H-O) and heart-diabetes (H-D) pathways.

## Discussion

Apart of structural network analysis, understanding the relationship between network position and biological function is of key importance. For mediators between heart diseases and obesity, we have found several enriched pathway terms related to diseases such as 'insulin signaling pathway', 'aspirin blocks signaling pathway involved in platelet activation' and 'natural killer cell-mediated cytotoxicity'. 'Kidney normal epithelium 3^rd^' is an example for associated tissues for H-O mediator proteins. The domains contained in these proteins, such as S-Tck, SH2 and SH3, are important domains in performing protein functions and are known to be involved in different pathogenic mechanisms [20,21].

The same procedure was used to explore functional information on H-D mediators. There are three enriched terms concerning pathways ('chemokine families', 'long-term depression' and 'cytokine-cytokine receptor'). Among these, 'chemokine families' are considered mostly pro-inflammatory: they can be induced during immune response to promote immune cells to the infection site, while others are considered homeostatic and are involved in controlling cell migration during normal tissue development. H-D mediators are mostly located in tissues 'adrenal tumor disease 3^rd^', 'pancreatic tumor disease 3^rd^' and contain domains like 'small inducible chemokine C/CC types' and 'small inducible cytokine A2 type'. The above analyses reveal generally pathogenic roles of H-O and H-D mediators. The following sections discuss some specific proteins of interest in order to demonstrate the potential use of our approach in systems biology and systems-based medicine.

We have found that the strongest indirect effect was mediated by P63104. This protein is the second ranked mediator for both H-O and H-D by M^2 ^index. This finding is in quite a concert with the spectrum of crucial functions of P63104 in the cell. P63104 is indeed an adapter protein implicated in the regulation of a large spectrum of both general and specialized signaling pathways. As a result, it binds to a large number of partners, usually by recognizing phosphoserine or phosphothreonine motifs. Its binding generally results in the modulation of the activity of the partners [22].

Another interesting mediator is C protein P12931, which mediate both H-O and H-D pathways. Since P12931 is involved in colorectal cancer [23], its centrality (based on any measure) is not surprising. P12931 (SRC_HUMAN) is the proto-oncogene tyrosine-protein kinase Src, belonging the protein kinase superfamily, Tyr protein kinase family and SRC subfamily. P12931 contains one protein kinase domain, one SH2 domain and one SH3 domain. This tyrosine-protein kinase plays its role in various signal transduction pathways in the cell, such as signaling by NGF, homeostasis, signaling in the immune system, signaling by EGFR as well as in the "gap junction trafficking and regulation" process.

P12931 mediates two types of disease proteins (P08588 (H), P16671 (H), P07550 (O) and P13945 (O)). Looking at the functionality of P16671 (CD36_HUMAN), P08588 (ADRB1_HUMAN), P07550 (ADRB2_HUMAN; the most influenced O protein) and P13945 (ADRB3_HUMAN), we see that these proteins all have receptor functions. Therefore, indirect effects of disorders may be performed by P12931 in cell signaling.

In addition, we have studied the most influenced proteins of heart diseases, obesity, and diabetes. The most influenced heart disease protein, P18825 (ADA2C_HUMAN) was found to synergistically influence the ejection fraction response to beta-blocker therapy of heart failure patients [24]. P18825 is an alpha-2 adrenergic receptor, mediating the catecholamine-induced inhibition of adenylate cyclase through the action of G proteins, belonging to the G-protein coupled receptor 1 family. P18825 relates to the activation of MAPK activity and cell-cell signaling. Alpha-2-adrenergic receptors have a critical role in regulating neurotransmitter release from sympathetic nerves and from adrenergic neurons in the central nervous system.

The most influenced diabetes protein was P51681 (CCR5_HUMAN), C-C chemokine receptor type 5, a receptor for a number of inflammatory CC-chemokines including MIP-1-alpha, MIP-1-beta and RANTES. Its signal transduction is based on increasing the intracellular calcium ion level. The genetic variation in CCR5 is associated with susceptibility to insulin-dependent diabetes mellitus type 22 (IDDM22, [25]). IDDM is caused by the body's own immune system which destroys the insulin-producing beta cells in the pancreas. Classical features are polydipsia, polyphagia and polyuria, due to hyperglycemia-induced osmotic diuresis. Like P12931, P51681 is involved in the interaction between the host cell's macromolecular machinery and viral proteins.

The most influenced O protein is P07550 (ADRB2_HUMAN), Beta-2 adrenergic receptor. Beta-adrenergic receptors mediate the catecholamine-induced activation of adenylate cyclase through the action of G proteins. P07550 belongs to the group of disulfide bond, glycoprotein, lipoprotein, palmitate and phosphoprotein. The findings of Large et al. [26] suggested that genetic variation in the ADRB2 gene might be of major importance for obesity, energy expenditure, and lipolytic ADRB2 function in adipose tissue, at least in women [26]. Tsai et al. [27] reported a significant association between a -47C-T polymorphism (arg-19cys; R-19C) in the beta-upstream peptide of the ADRB2 gene and bronchodilator drug response among 264 African American children with asthma [27].

## Conclusions

We illustrate that understanding the role and importance of individual genes highly depends on how to define the network of study: proteins appearing as hubs in the PPI network may be of lower topological importance in mediating indirect effects among groups of disease proteins (in the DPIP network, see [28]). By quantitatively identifying, the most important mediator genes are seemingly unimportant but play key roles in maintaining communication between disease genes and these mediators are not hubs in PPI. We suggest that the central proteins in the IP network can be even more important in systems-based medicine and drug design than either the hubs or the disease proteins themselves. The proteins mediating indirect interactions among the studied disease proteins were found to be generally of high functional importance in the cell (for example P63104). Even if obesity and diabetes-related proteins are not directly linked to heart-disease proteins, rich indirect linkages can be realized through, for example, the insulin signaling pathway or the P12931 proto-oncogene tyrosine-protein kinase Src. We propose that our results should call for experimental studies on the relevance of this approach.

## Methods

### Data

We investigated two main databases: the i2d database as a comprehensive human protein interaction database (formerly known as OPHID [29]), and the OMIM database as a well-known disease gene database [30]. The i2d database is an on-line database of known and predicted mammalian and eukaryotic protein-protein interactions. It consists of almost all human protein interaction data sets (including HRPD, BIND, etc), and this is the reason why we chose the i2d database for our analysis. To obtain a more reliable set of protein interactions, we excluded all the interactions obtained by homology methods. Identifier (ID) of protein in the i2d database is the protein ID defined in the UniProt database [31]. The OMIM database is a catalog of human genes and genetic disorders. In OMIM, the list of hereditary disease genes is described in the OMIM morbid map. To extract the list of genes related to five, we searched for all genes having the keywords "cancer", "heart disease", "diabetes", "autism" and "obesity". Our study considered the database version released in January 2009. By using a mapping scheme, product proteins, so-called disease proteins corresponding to the disease genes in the OMIM database, are identified by UniProt protein IDs. We then did a preprocess procedure to filter noise data (for both i2d-based PPI data and OMIM-based disease protein data). We note that improved databases will probably influence the actual results but considering also another disease in the same database does not. Characterizing the linkage between heart disease and obesity proteins, for example, is not influenced by analyzing some additional kind of disease proteins.

Based on these data sets, we have constructed (1) a human protein-protein interaction network (PPI), (2) a network of disease proteins (DP) causing five selected diseases plus their interacting partners (IP; called the DPIP network) and (3) a protein network of IPs with interactions among them (IP network, see Figure 1). The DPIP and the IP networks are subgraphs of the PPI network. The IP network is not a subgraph of the DPIP network, since it contains IP-IP interactions not included in the DPIP network - the network of only DP-DP and DP-IP interactions.

After preprocessing, the PPI network contained 12513 nodes and 60675 links. In the DPIP network, there were 2777 nodes (9 H, 9 A, 20 D, 44 O, 90 C and 2349 IP proteins). Most pairs of diseases had also direct links between their protein sets (like some A proteins with C proteins and some A proteins with D proteins), but four pairs (H and O, H and D, H and A and O and A) were only indirectly linked (mostly through IPs, but rarely also through other disease proteins, predominantly C). Links in each network were considered undirected and un-weighted. We considered only two-step-long indirect interactions between proteins (note that longer pathways link each pair of nodes in a network, but longer indirect effects may be of less practical relevance).

### Network analysis

One of the key interests of systems biology is to understand the components of biological systems in a wider, non-local context. In a network model, this means the analysis of network nodes also from the viewpoint of indirect linkages and determination. Various centrality indices provide a rich toolkit for quantifying the non-local neighborhood of graph nodes in directed and undirected (symmetrical), binary and weighted as well as signed and unsigned networks. In particular, we use here local (node degree), non-local (betweenness centrality) and non-local, short-range (topological importance in two steps) network measures.

Here we were interested in finding the proteins of central positions in the network, as they can be highly important also from a functional point of view (see [32]). A graph model of a biological system is composed of N nodes and M links connecting them, where nodes and links can represent proteins and protein interactions, for example. The number of neighbors for node *i *(D_i_, its degree) provides a quick look at how richly a protein is linked to others: high-degree nodes are called hubs and can be considered of high structural importance. Normalized degree (nD_i_) is suitable for analyses where there are networks of different size to analyze:(1)

where N is the number of nodes in the network and auto-loops are not considered (Additional file 4 shows the nD values for all nodes in each of the three studied networks, as well as their ranks).

Degree is a local network metric, but other centrality measures are capable of providing additional information, for example, also on the number of neighbors of neighbors (i.e. how richly the neighbors are linked). There is a rising interest in indirect network measures in molecular and cell biology, e.g. [33,34], following other fields of biology. For example, the betweenness centrality of node *i *(B_i_, [35]) measures how frequently node *i *is incident to all shortest paths between the other pairs of nodes *j *and *k *in the network:(2)

where *i *≠ *j *≠ *k*, *g*_*jk *_is the number of equally shortest paths between nodes *j *and *k*, and *g*_*jk*_(*i*) is the number of these shortest paths which node *i *is incident to. For studying different networks, the normalized value (nB_i_) is suitable:(3)

where N is the number of nodes in the network and auto-loops are not considered (Additional file 4 shows the nB values for all nodes in each of the three studied networks, as well as their ranks).

Another index measuring indirect neighborhood of graph nodes is the topological importance (TI_i _^n^) index [16,17]. It is suitable if the topological relationship between particular nodes *i *and *j *are to be quantified, even if *i *and *j *are not linked: the TI_i _^n ^index quantifies the expected strength of indirect effects between them, mediated by one or several mediator node(s). If *i *and *j *are linked through *k*, it can also be quantified how large is the contribution of *k *to connecting *i *and *j *(relative to other pathways of shorter, equal or longer length).

We assume a network with undirected links where effects can spread in any direction with the same probability. Here, we define a_ij _^n ^as the effect of node *j *on node *i *when *i *can be reached from *j *in exactly *n *steps. The simplest mode of calculating a_ij _^n ^is when *n *= 1 (i.e. the effect of node *j *on node *i *in 1 step):(4)

where D_i _is the degree of node *i *(i.e. the number of its neighbors). We assume that indirect effects are multiplicative and additive: if we wish to determine the effect of node *j *on node *i *in two steps, and there are two such two-step pathways (one through *k *and the other through *f*), then the effects of *j *on *i *through *k *is defined as the product of two direct effects:(5)

(therefore the term multiplicative), and similarly, the effect of *j *on *i *through *f *equals(6)

Further, to determine the overall two-step effect of *j *on *i *(a_ij _^2^), we simply sum up the effects mediated by different two-step pathways (through *k *and through *f*):(7)

(therefore the term additive). Now we are interested only in two-step long pathways but this method can also be used for longer indirect pathways (also in weighted graphs, see [17]). A software called SilInd 1.2 (code available from Dr. Liu: wliu56@gate.sinica.edu.tw) can calculate these indices, so effects can be measured for single a_ij _interactions and a_ij(k) _pathways, as well as the total topological importance of graph node *j *can be measured as the sum of its effects on other nodes:(8)

where *N *is the number of nodes in the network and effects are mediated through *n*-step pathways.

Based on this, it can be determined how the proteins of a certain disease (disease 1, D1) are topologically related to proteins of another disease (disease 2, D2):(9)

**Figure 7:**
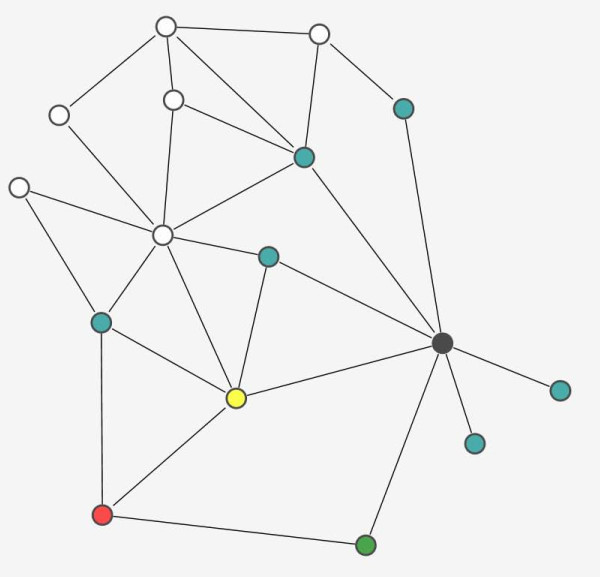
**A toy network illustrating how to measure indirect effects**. In this toy PPI network, there are two disease proteins, a red (D degree = 3) and a black (D = 7) one. Their interacting partners (IPs) are colorful: the green (D = 2) and the yellow (D = 5) nodes mediate two-step-long indirect effects between disease proteins, the blue ones do not. White nodes are not IPs. The PPI network contains all nodes, the DPIP network contains all colorful nodes but no links among IPs, while the IP network contains only IPs and the links among them. The strength of the effect from the red node to the green one is 1/2, and from the green to the black node it equals 1/7, thus, the red-green-black pathway is of strength 1/14. Similarly, the red-yellow-black pathway is of strength 1/5*1/7 = 1/35. Additively, the two-step-long indirect effects from red to black equal 1/14+1/35 = 49/490 = 1/10. From black to red, the strength of two-step-long indirect influence is (1/5*1/3)+(1/2*1/3) = 7/30. The summed mutual dependence equals 7/30+1/10 = 10/30 = 1/3. The relative contribution of the two mediator proteins are 1/15+1/35 = 50/525 = 0.095 (yellow) and 1/14+1/6 = 20/84 = 5/21 = 0.238 (green). Figure drawn by [38].

A^n ^_D1;D2 _is zero if there are no proteins involved in causing "disease 1" that is linked (within *n *steps) to any of the proteins involved in "disease 2". Higher values mean several *n*-step-long pathways between the two sets of proteins being involved in the two diseases. The role of a single protein *k *in mediating a two-step long pathway between *i *and *j *can also be quantified:(10)

that quantifies mediated effects in both directions (see also Figure 7 for explanation).

Apart of the structural analysis of proteins in the PPI, DPIP and IP networks, we were also interested in the biological functions of most important mediator proteins (measured by M^2 ^in the DPIP network, see Additional file 3). We analysed the 11 H-O mediators and, accordingly, the 11 most important ones for H-D pathways (out of 24). We studied their pathways, tissues and GO terms extracted from the DAVID database system ([36,37], http://david.abcc.ncifcrf.gov/). The DAVID database is a high-throughput and integrated data-mining environment to systematically study gene lists derived from high-throughput genomic experiments. Additional files 5 and 6 show information on main GO categories (MF: molecular function, BP: biological process and CC: cellular component), GO terms (e.g. cell death), count (how many proteins out of 11 are characterized by the given GO term), the P-value of the GO term in the database and, finally, the list of the proteins counted. Additional files 5 and 6 provide information on mediators in H-O and H-D pathways, respectively. Each mediator protein is characterized by the average P-value of all GO terms characterizing it (see Table 4). The relationships between M^2 ^index and the average P-value calculated for GO terms of the mediators in H-O and H-D are then measured by Pearson and Spearman rank order correlation by using Free Statistics Software, Office for Research Development and Education, version 1.1.23-r6 http://www.wessa.net/.

**Table 4:** P-values of most important H-O and H-D mediators.

H-O protein	avP	H-D protein	avP
Q5JY77	0.0454	P13500	0.0633
P63104	0.0453	P05129	0.0590
O14908	0.0405	P07948	0.0511
Q14232	0.0356	P22681	0.0370
P17252	0.0345	P09471	0.0342
P28482	0.0328	P63104	0.0282
P49407	0.0305	P80098	0.0271
P06241	0.0269	P80075	0.0234
P62993	0.0244	Q99962	0.0198
P41240	0.0220	Q99616	0.0153
P54646	0.0191	P48745	0.0120

The average P-values (avP) of the most important 11 mediator proteins in the heart-obesity (H-O) and heart-diabetes (H-D) pathways. Note that smaller P-values mean functionally more important proteins.

## Abbreviations

DPIP NETWORK: the network of Disease Proteins plus their Interacting Partners; I2D: Interologous Interaction Database; IP NETWORK: the network of Interacting Partners of disease proteins; OMIM: Online Mendelian Inheritance in Man; UNIPROT: The universal protein resource.

## Authors' contributions

TPN suggested the key idea, analyzed the database and wrote the paper. FJ made network analysis and wrote the paper. All authors read and approved the final manuscript.

## Supplementary Material

Additional file 1 **Identity of mediator proteins among H proteins causing heart diseases (in columns) and D proteins causing diabetes (in rows)**. Most of the mediators are IP proteins (in black), there is a single disease protein connecting the other two sets of disease proteins (P12931, belonging to C, in red). Click here for file

Additional file 2 **The relative strength of two-step-long indirect interactions (A_H,D_^2^) mediated by mediators among H and D**. Column sums would give which H protein is mostly influenced by D proteins (P18825), while row sums would give which D protein is mostly influenced by H proteins (P51681). The largest value (0.17983) indicates the strongest indirect effect between the two diseases (corresponding to P09471, see Additional file 1). Click here for file

Additional file 3 **The most important mediator proteins are shown among H and O as well as H and D sets of disease proteins, ranked according to their mediation strength M^2 ^(calculated for the DPIP network)**. Click here for file

Additional file 4 **the values and ranks of nD and nB values for the nodes of the PPI, DPIP and IP networks. Also, the nD vs nB relationships and the value rank column diagrams are given for each network**. Click here for file

Additional file 5 **the count, P-value and identity of H-O mediator proteins chracterized by particular GO categories and terms**. Click here for file

Additional file 6 **the count, P-value and identity of H-D mediator proteins chracterized by particular GO categories and terms**. Click here for file
