# Supplementary material for: A quantitative approach to study indirect effects among disease proteins in the human protein interaction network
Source: BMC Syst Biol. 2010 Jul 29;4:103. doi: 10.1186/1752-0509-4-103 (PMC2924296; doi:10.1186/1752-0509-4-103)
Supplement: Additional file 1 — Identity of mediator proteins among H proteins causing heart diseases (in columns) and D proteins causing diabetes (in rows). Most of the mediators are IP proteins (in black), there is a single disease protein connecting the other two sets of disease proteins (P12931, belonging to C, in red). [file 1752-0509-4-103-S1.DOCX]

| D \ H | P08254 | P08588 | P16671 | P17302 | P18825 | P78504 | Q14524 | Q9UGJ0 | Q9Y4J8 |
| --- | --- | --- | --- | --- | --- | --- | --- | --- | --- |
| O14901 |  |  |  | **P28482** |  |  |  |  |  |
| O76024 |  |  |  |  |  |  |  |  |  |
| P00995 |  |  |  |  |  |  |  |  |  |
| P01185 |  |  |  |  |  |  |  |  |  |
| P01308 |  |  |  | **P48745** |  |  |  |  |  |
| P01589 |  |  |  |  |  |  |  |  |  |
| P05231 |  | **Q99962** |  |  |  |  |  |  |  |
| P06213 |  |  | **P12931** | **P41240 P17252 P27361 Q03135 P12931** |  |  | **P22681** |  |  |
| P11150 |  |  |  |  |  |  |  |  |  |
| P11168 |  |  |  |  |  |  |  |  |  |
| P11226 |  |  |  |  |  |  |  |  |  |
| P14672 |  |  |  |  |  |  |  |  |  |
| P16410 |  |  | **P07948 P06241** |  |  |  |  |  |  |
| P19835 |  |  |  |  |  |  |  |  |  |
| P20823 |  |  | **P12931** | **P12931** |  |  | **P02768** |  |  |
| P22413 |  |  |  |  |  |  |  |  |  |
| P30518 |  |  |  |  |  |  |  |  |  |
| P31751 | **O14788** |  | **P12931** | **P12931** | **P63104** |  |  |  |  |
| P35557 |  |  |  |  |  |  |  |  |  |
| P35568 |  | **P62993** | **P06241** | **P05129 P28482 Q03135** | **P63104** |  |  |  |  |
| P35680 |  |  |  |  |  |  |  |  |  |
| P41181 |  |  |  |  |  |  |  |  |  |
| P41235 |  |  |  |  |  |  |  | **P54646** |  |
| P43304 |  |  |  |  |  |  |  |  |  |
| P47871 |  |  |  |  |  |  |  |  |  |
| P51681 | **P80075 P13500 P80098 Q99616** | **P49407** |  |  | **P09471** |  |  |  |  |
| P52945 |  |  |  |  |  |  |  |  |  |
| Q09428 |  |  |  |  |  |  |  |  |  |
| Q13562 |  |  |  |  |  |  |  |  |  |
| Q14573 |  |  |  |  |  |  |  |  |  |
| Q14654 |  |  |  |  |  |  |  |  |  |
| Q6EEV6 |  |  |  |  |  |  |  |  | **P62736** |
| Q7RTS3 |  |  |  |  |  |  |  |  |  |
| Q8NEA6 |  |  |  |  |  |  |  |  |  |
| Q9BYX4 |  |  |  |  |  |  |  |  |  |
| Q9BZS1 |  |  |  |  |  |  |  |  |  |
| Q9HC96 |  |  |  |  |  |  |  |  |  |
| Q9HD89 |  |  |  |  |  |  |  |  |  |
| Q9NQB0 |  |  |  |  |  |  |  |  |  |
| Q9UM63 |  |  |  |  |  |  |  |  |  |
| Q9UQF2 |  |  | **P05106** |  |  |  |  |  |  |
| Q9UQQ2 |  | **P62993** |  |  |  |  |  |  |  |
| Q9Y2R2 |  | **P62993** |  | **P41240** |  |  | **P22681** |  |  |
| Q9Y4H2 |  | **P62993** |  |  | **P63104** |  |  |  |  |

**Additional file 1**: Identity of mediator proteins among H proteins causing heart diseases (in columns) and D proteins causing diabetes (in rows). Most of the mediators are IP proteins (in black), there is a single disease protein connecting the other two sets of disease proteins (P12931, belonging to C, in red).
