# Supplementary material for: A quantitative approach to study indirect effects among disease proteins in the human protein interaction network
Source: BMC Syst Biol. 2010 Jul 29;4:103. doi: 10.1186/1752-0509-4-103 (PMC2924296; doi:10.1186/1752-0509-4-103)
Supplement: Additional file 2 — The relative strength of two-step-long indirect interactions (AH,D2) mediated by mediators among H and D. Column sums would give which H protein is mostly influenced by D proteins (P18825), while row sums would give which D protein is mostly influenced by H proteins (P51681). The largest value (0.17983) indicates the strongest indirect effect between the two diseases (corresponding to P09471, see Additional file 1). [file 1752-0509-4-103-S2.DOCX]

| D \ H | P08254 | P08588 | P16671 | P17302 | P18825 | P78504 | Q14524 | Q9UGJ0 | Q9Y4J8 |
| --- | --- | --- | --- | --- | --- | --- | --- | --- | --- |
| O14901 |  |  |  | **0.02404** |  |  |  |  |  |
| O76024 |  |  |  |  |  |  |  |  |  |
| P00995 |  |  |  |  |  |  |  |  |  |
| P01185 |  |  |  |  |  |  |  |  |  |
| P01308 |  |  |  | **0.06066** |  |  |  |  |  |
| P01589 |  |  |  |  |  |  |  |  |  |
| P05231 |  | **0.07738** |  |  |  |  |  |  |  |
| P06213 |  |  | **0.00037** | **0.03575** |  |  | **0.01339** |  |  |
| P11150 |  |  |  |  |  |  |  |  |  |
| P11168 |  |  |  |  |  |  |  |  |  |
| P11226 |  |  |  |  |  |  |  |  |  |
| P14672 |  |  |  |  |  |  |  |  |  |
| P16410 |  |  | **0.06428** |  |  |  |  |  |  |
| P19835 |  |  |  |  |  |  |  |  |  |
| P20823 |  |  | **0.00047** | **0.00042** |  |  | **0.02102** |  |  |
| P22413 |  |  |  |  |  |  |  |  |  |
| P30518 |  |  |  |  |  |  |  |  |  |
| P31751 | **0.03217** |  | **0.02177** | **0.0003** | **0.03421** |  |  |  |  |
| P35557 |  |  |  |  |  |  |  |  |  |
| P35568 |  | **0.00512** | **0.00925** | **0.05289** | **0.03489** |  |  |  |  |
| P35680 |  |  |  |  |  |  |  |  |  |
| P41181 |  |  |  |  |  |  |  |  |  |
| P41235 |  |  |  |  |  |  |  | **0.03502** |  |
| P43304 |  |  |  |  |  |  |  |  |  |
| P47871 |  |  |  |  |  |  |  |  |  |
| P51681 | **0.16374** | **0.02444** |  |  | **0.17983** |  |  |  |  |
| P52945 |  |  |  |  |  |  |  |  |  |
| Q09428 |  |  |  |  |  |  |  |  |  |
| Q13562 |  |  |  |  |  |  |  |  |  |
| Q14573 |  |  |  |  |  |  |  |  |  |
| Q14654 |  |  |  |  |  |  |  |  |  |
| Q6EEV6 |  |  |  |  |  |  |  |  | **0.01524** |
| Q7RTS3 |  |  |  |  |  |  |  |  |  |
| Q8NEA6 |  |  |  |  |  |  |  |  |  |
| Q9BYX4 |  |  |  |  |  |  |  |  |  |
| Q9BZS1 |  |  |  |  |  |  |  |  |  |
| Q9HC96 |  |  |  |  |  |  |  |  |  |
| Q9HD89 |  |  |  |  |  |  |  |  |  |
| Q9NQB0 |  |  |  |  |  |  |  |  |  |
| Q9UM63 |  |  |  |  |  |  |  |  |  |
| Q9UQF2 |  |  | **0.03113** |  |  |  |  |  |  |
| Q9UQQ2 |  | **0.01008** |  |  |  |  |  |  |  |
| Q9Y2R2 |  | **0.0091** |  | **0.0162** |  |  | **0.025** |  |  |
| Q9Y4H2 |  | **0.00567** |  |  | **0.03583** |  |  |  |  |

**Additional file 2**: The relative strength of two-step-long indirect interactions (A_H,D_^2^) mediated by mediators among H and D. Column sums would give which H protein is mostly influenced by D proteins (P18825), while row sums would give which D protein is mostly influenced by H proteins (P51681). The largest value (0.17983) indicates the strongest indirect effect between the two diseases (corresponding to P09471, see Additional file 1).
