# Supplementary material for: A quantitative approach to study indirect effects among disease proteins in the human protein interaction network
Source: BMC Syst Biol. 2010 Jul 29;4:103. doi: 10.1186/1752-0509-4-103 (PMC2924296; doi:10.1186/1752-0509-4-103)
Supplement: Additional file 3 — The most important mediator proteins are shown among H and O as well as H and D sets of disease proteins, ranked according to their mediation strength M2 (calculated for the DPIP network). [file 1752-0509-4-103-S3.DOCX]

**Additional file 3:** The most important mediator IP proteins are shown among H and O as well as H and D sets of disease proteins, ranked according to their mediation strength M^2^ (calculated for the DPIP network). The most important shared mediator is in red.

|  |  |  |  |
| --- | --- | --- | --- |
| O-H mediators |  | D-H mediators |  |
| protein | M^2^ | protein | M^2^ |
| Q14232 | 0,12301 | P09471 | 0,17983 |
| P63104 | 0,11666 | P63104 | 0,10493 |
| O14908 | 0,05745 | Q99962 | 0,07738 |
| Q5JY77 | 0,05357 | P48745 | 0,06066 |
| P54646 | 0,05079 | P07948 | 0,05128 |
| P06241 | 0,02742 | P80075 | 0,0429235 |
| P49407 | 0,026785 | P13500 | 0,0429235 |
| P41240 | 0,0264 | P80098 | 0,0429235 |
| P17252 | 0,01091 | Q99616 | 0,0429235 |
| P28482 | 0,00707 | P05129 | 0,0391865 |
| P62993 | 0,006302 | P22681 | 0,03839 |
|  |  | P54646 | 0,03502 |
|  |  | O14788 | 0,03217 |
|  |  | P05106 | 0,03113 |
|  |  | P28482 | 0,0300687 |
|  |  | P62993 | 0,02997 |
|  |  | P41240 | 0,0246877 |
|  |  | P06241 | 0,0246346 |
|  |  | P49407 | 0,02444 |
|  |  | P02768 | 0,02102 |
|  |  | Q03135 | 0,015476 |
|  |  | P62736 | 0,01524 |
|  |  | P27361 | 0,0109127 |
|  |  | P17252 | 0,0084877 |
